# An Applied Framework for Incorporating Multiple Sources of Uncertainty in Fisheries Stock Assessments

**DOI:** 10.1371/journal.pone.0154922

**Published:** 2016-05-10

**Authors:** Finlay Scott, Ernesto Jardim, Colin P. Millar, Santiago Cerviño

**Affiliations:** 1 European Commission, Joint Research Centre (JRC), Institute for the Protection and Security of the Citizen (IPSC), Maritime Affairs Unit, via Enrico Fermi 2749, 21027 Ispra (VA), Italy; 2 Marine Scotland, Freshwater Laboratory, Faskally, Pitlochry, PH16 5LB, United Kingdom; 3 Instituto Español de Oceanografía, Centro Oceanográfico de Vigo, Subida a Radio Faro 50, 36390 Vigo, Spain; Aristotle University of Thessaloniki, GREECE

## Abstract

Estimating fish stock status is very challenging given the many sources and high levels of uncertainty surrounding the biological processes (e.g. natural variability in the demographic rates), model selection (e.g. choosing growth or stock assessment models) and parameter estimation. Incorporating multiple sources of uncertainty in a stock assessment allows advice to better account for the risks associated with proposed management options, promoting decisions that are more robust to such uncertainty. However, a typical assessment only reports the model fit and variance of estimated parameters, thereby underreporting the overall uncertainty. Additionally, although multiple candidate models may be considered, only one is selected as the ‘best’ result, effectively rejecting the plausible assumptions behind the other models. We present an applied framework to integrate multiple sources of uncertainty in the stock assessment process. The first step is the generation and conditioning of a suite of stock assessment models that contain different assumptions about the stock and the fishery. The second step is the estimation of parameters, including fitting of the stock assessment models. The final step integrates across all of the results to reconcile the multi-model outcome. The framework is flexible enough to be tailored to particular stocks and fisheries and can draw on information from multiple sources to implement a broad variety of assumptions, making it applicable to stocks with varying levels of data availability The Iberian hake stock in International Council for the Exploration of the Sea (ICES) Divisions VIIIc and IXa is used to demonstrate the framework, starting from length-based stock and indices data. Process and model uncertainty are considered through the growth, natural mortality, fishing mortality, survey catchability and stock-recruitment relationship. Estimation uncertainty is included as part of the fitting process. Simple model averaging is used to integrate across the results and produce a single assessment that considers the multiple sources of uncertainty.

## Introduction

Stock assessment can be defined as the application of quantitative and statistical models to estimate the current and historical status and trends of a fish stock, including abundance, mortality and productivity [1]. A more recent definition used by the World Conference on Stock Assessment Methods (WCSAM), is “Stock assessment is the synthesis of information on life history, fishery monitoring, and resource surveys for estimating stock size and harvest rate relative to sustainable reference points. … Stock assessment is usually carried out by applying mathematical models that fit available information to provide simplified representations of population and fishery dynamics.” [2].

Fisheries management is increasingly focused on the management of risk [3]. For a stock assessment to be included as part of a risk-based approach to fisheries management it is necessary for the assessment to consider multiple sources of uncertainty. Six types of uncertainty have been identified as important sources of risk in a fisheries setting [4]. In this study we focus on three of them: *process*, stochasticity in the population dynamics arising from natural variability in demographic rates; *model*, arising from lack of information about the correct conceptual model, including model structure, parameters and error structure; and *estimation*, uncertainty in the estimated parameters as a result of the model fitting process. The remaining three types of uncertainty: *observation*, arising from data collection, measurement and sampling; *implementation*, how well a management policy is fulfilled and *institutional*, arising from interactions between different groups of people (e.g. scientists and fishermen) are not explored in this study. This does not mean that they are unimportant in the context of fisheries management but that they are usually used in the testing of management options, notably in management strategy evaluation (MSE) algorithms which is outside the scope of this paper.

Typically, stock assessment only considers estimation uncertainty, for example, through the use of confidence intervals on the estimated values. This means that the resulting uncertainty is underestimated leading to advice that may be insufficently robust. For example, assumptions on natural mortality can have a strong impact on the outcome of a stock assessment, particularly on the estimates of fishing mortality. There is a large degree of uncertainty on natural mortality and how it may change with length or age, partly because it is very difficult to measure. Despite this, stock assessments seldom consider the uncertainty in natural mortality and often a single value is used for all lengths or ages and years.

Combining multiple sources of uncertainty can be used to generate a suite of candidate stock assessments that reflect the different underlying assumptions made about the stock. However, often only a single ‘best’ stock assessment is then selected from the suite, e.g. [5]. This has two main issues. The first is deciding how that single assessment is selected, which can be done through a combination of quantitative (e.g. calculating AIC or other metrics) and qualitative (e.g. inspecting residuals) approaches. The second is that by selecting a single assessment, all of the other plausible assessments and their accompanying uncertainty are rejected, ignoring what may be relevant representations of reality.

An alternative to selecting a single stock assessment is to integrate across all of the results and their uncertainties into a final outcome. Several methods are available to do this including model averaging, a technique for incorporating model-selection uncertainty into inference [6]. It can be thought of as a model-weighting algorithm where the weights are based on the support for the model in the data and where each model represents a different, plausible hypotheses. A variety of model averaging approaches have been proposed: frequentist and Bayesian, simple and complex [7]. One of the key questions is how to weight the models when averaging over them [8].

Generating the suite of candidate assessments is made more straightforward through the use of a flexible stock assessment framework that can include multiple sources of uncertainty. The *assessment for all* (*a4a*) initiative presents has been developed to allow uncertainty about biological processes such as growth and natural mortality to be taken into account and their uncertainties propagated through to the estimates of population abundance, fishing mortality and reference points by the stock assessment model [9]. To develop a range of assumptions on the biological processes the *a4a* approach encourages the use of information from diverse sources such as scientific papers, Ph.D. theses, Fishbase, other stocks, etc., and also the use of generic information on life history invariants to derive generic priors, as suggested by [10]. As well as considering uncertainty about the biology of the stock, the *a4a* approach also facilitates the inclusion of uncertainty about the stock assessment model, for example through the use of different models of selectivity, or different assumptions on survey catchability, etc. This approach is different to other stock assessment models such as XSA [11] which are rigid in their approach and use a fixed set of assumptions.

The assessment of the Iberian hake stock (*Merluccius merluccius*) in ICES Divisions VIIIc and IXa (FAO Area 27) has been carried out by ICES since the mid 90’s [12]. As in many other assessments, the models used to carry out the evaluation of the status of the stock have changed during this time and have included an XSA model [11], a Bayesian catch-at-age model [13] and, more recently, a Gadget [14] model to account for the recent changes in the perception of hake growth [12, 15].

Accounting for the uncertainty associated with the assessment has always been a concern, and the enforcement of a recovery plan in 2005 (Reg. EC No 2166/2005) made it more urgent to tackle the problem. Several studies on this subject were performed to explore alternative assumptions about the estimation of discards [13, 16], reproduction and productivity [17] and growth [18]. The Gadget model limits the possibilities of fully exploring uncertainty and does not allow the calculation of estimation uncertainty, partly due to the slow convergence which makes it impractical to use simulations to derive statistical properties of the parameters, and partly due to the characteristics of the minimiser that does not always accurately estimate the Hessian matrix from which to derive a variance-covariance matrix for the parameters.

The objectives of this work are to use the tools developed under the *a4a* framework to (i) develop a method to integrate distinct sources of uncertainty in a stock assessment and use simple model averaging to combine the results in a coherent dataset, which can be used for advice; and (ii) test the methodology on the Iberian hake stock. We show that it is straightforward to include uncertainty from a wide range of sources (biological parameters, biological models, stock assessment models and model fit) in a stock assessment, thereby better accounting for the overall uncertainty in the results leading to the provision of more robust advice.

## Materials and Methods

This study focuses on introducing several types and sources of uncertainty in the stock assessment process for Iberian hake (ICES Divisions VIIIc and IXa), starting with the initial length-based survey and fisheries data and resulting in estimates of age-based abundance and fishing mortality. As mentioned in the introduction, we focus on including *process*, *model* and *estimation* uncertainty. These types of uncertainty are included at different stages in the approach and are propagated through to the final result (Table 1).

**Table 1:** Sources and types of uncertainty included in this stock assessment process.

Source	Type
Variance in growth parameters	Process
Variance in natural mortality parameters	Process
Natural mortality model choice	Model
Stock assessment submodel choice	Model
Resampling from stock assessment fit	Estimation

The approach introduces different types and sources of uncertainty into the stock assessment process. These uncertainties are propagated through to the final results.

**Fig 1:**
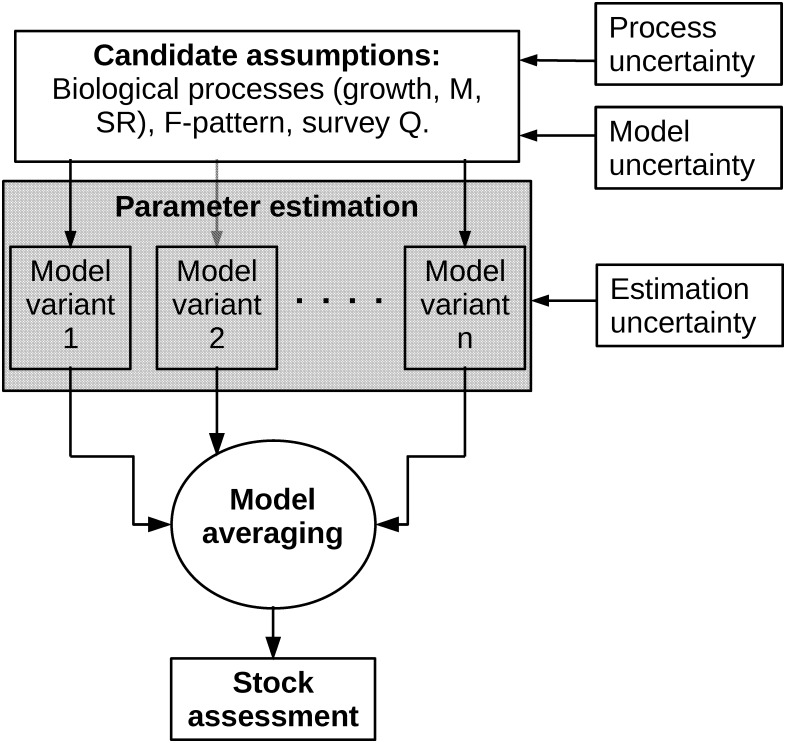
Schematic diagram of the approach. The approach has three steps: Generating the candidate assumptions, estimating the parameters of the stock assessment models and averaging across the model variants. Process and model uncertainty are introduced when generating the candidate assumptions. Estimation uncertainty is introduced during parameter estimation. The result is a single stock assessment that integrates across the multiple sources of uncertainty. *M* is natural mortality, *SR* is the stock-recruitment relationship, *F-pattern* is the fishing mortality pattern, *survey Q* is the survey catchability pattern.

The approach has three steps (Fig 1). The first step involves generating and conditioning a range of candidate assumptions about the stock including on the biological processes, fishing mortality, survey catchability and stock-recruitment relationship. For Iberian hake the biological processes of interest are growth and natural mortality which are considered to be highly uncertain. It would also be possible to consider other biological processes such as maturity. Combinations of the candidate assumptions can be considered as alternative, plausible states of nature. These assumptions contain different sources and types of uncertainty. Process uncertainty was introduced through the biological parameters of the growth and natural mortality models. Biological model uncertainty was introduced through the use of two alternative natural mortality models. Further model uncertainty was introduced through the use of a range of stock assessment models. The second step involves the estimation of the unknown model parameters, such as the fishing mortality and stock abundance, by fitting the stock assessment models. Estimation uncertainty was generated from the fitting process by resampling from the fits. The outcome of this step was a suite of stock assessment results that consider multiple sources of uncertainty. For the final step, instead of choosing a single ‘best’ assessment the results are integrated to reconcile the multi-model outcomes and combine the different sources of uncertainty. Here we use a simple model averaging approach.

All analyses were carried out using R [19], FLR [20] and several R packages referenced in the relevant sections.

### Length-based data

The study used the stock data from 2014 [21]. This includes annual length-based catch, landings and discards abundances from 1982 to 2012, recorded in 1 cm length classes from 1 cm to 129 cm. Mean weights at length were calculated using *W* = *aL*^*b*^ where *a* = 6.59e-5 and *b* = 3.01721 [12].

Three length based indices of abundance covering the whole stock area were available: the Spanish October groundfish survey in the North of Spain (1983–2012); the Portuguese October groundfish in the Portuguese Atlantic coast (1989–2011) and the Gulf of Cadiz November survey in the South of Spain (1999–2012). The index data was binned into 2 cm length classes

A matury ogive based on data from annual Spanish sampling during the main spawning season (December to May) was used [12]. In this study the ogive is assumed to be constant in time and is taken as the mean of the last three years (2010–2012).

### Process uncertainty in the growth parameters

The stock assessment model used here (*a4a* [9]) is age-based making it necessary to convert the length-based indices and stock data to be age-based. This was done using a simple length-slicing method (see below) based on the von Bertalanffy growth equation [22] (the following methods are also appropriate for alternatives such as the Gompertz model [23]). This required the generation of values for the von Bertalanffy growth parameters *L*_∞_, *k* and *t*0.

Process uncertainty was introduced through the inclusion of variability in the growth parameters using a t-copula [24, 25] with triangle marginal distributions. Copulas allow for flexibility in multivariate distribution models, allowing for more robust distributions than the common multivariate gaussian. A triangle distribution is described by the minimum, maximum and median values of the distribution. It makes very few assumptions about the parameters and simplifies the definition of bounds, thereby ensuring that the sampled values of each parameter are within well specified limits. The parametrization of the copula requires an unscaled variance-covariance matrix and the limits and medians of the triangle marginals.

The marginal distributions of each parameter were set using minimum, maximum and median values (Table 2). The minimum and maximum values of *L*_∞_ and *k* were set to +- 1.96 standard deviations from the median (thereby covering approximately 95% of the variation), with a coefficient of variation of 10%. The median parameter values for *L*_∞_ and *k* were taken from the most recent assessment (130 cm and 0.164 y^-1^ respectively) [21]. The maximum value of *t*0 was set to 0 and the minimum value was set to the lowest value that gave a positive age at the smallest length (1 cm) given the ranges of *L*_∞_ and *k*. The median value of *t*0 was set so that the marginal distribution is a symmetrical triangle.

**Table 2:** Values of the marginal triangle distribution parameters for the von Bertalanffy growth parameters.

Parameter	Minimum	Median	Maximum
*L*_∞_ (*cm*)	104.520	130	155.480
*k* (*year*^−1^)	0.132	0.164	0.196
*t*0 (*year*)	-0.184	-0.092	0

The unscaled variance-covariance matrix was computed using data from Fishbase [26]. The number of records in Fishbase which had values for all three parameters that were not “questionable” (a qualitative Yes / No description in the Fishbase data to identify unreliable data) and that were only for hake was insufficient to reliably estimate the variance-covariance matrix (67 records). Instead the variance-covariance matrix was estimated using the records for all demersal species that had values for all three parameters and that were not questionable (2882 records) [10]. When generating the uncertainty using copulas, the variance-covariance matrix is effectively scaled by the variance on the individual parameters [24, 25] and does not determine the magnitude of the parameter uncertainty, only the relative uncertainty between the parameters. 1000 parameter sets were sampled from the multivariate distribution.

The distribution was evaluated using the R packages copula [27] and triangle [28].

### Model and process uncertainty in the natural mortality

In the current stock assessment for Iberian hake there is no uncertainty in the natural mortality assumptions and a fixed value of 0.4 is used for all ages and years [21]. Many different natural mortality models have been proposed that are based on biological and ecological theory [29–31]. Here, model uncertainty is introduced in the natural mortality assumptions through the use of two natural mortality models. Process uncertainty is then introduced through the parameters for one of the models.

The first model follows the current stock assessment and uses a fixed value of 0.4 for all lengths and years with no process uncertainty. This model is referred to as the ‘0.4’ model.

The second model is a length-based model where the shape of the natural mortality by length follows ‘Gislason’s Second Estimator’ [29, 32]:

$$m_{len}=k\;{\left(L_{\infty }/len\right)}^{1.5}$$ (1)

To set the absolute level of *m*_*len*_, the values are scaled so that the mean values over the lengths 15 to 60 cm, the most exploited lengths, are equal to ‘Jensen’s Second Estimator’ [29, 33]:

$$m_{av}=1.5k$$ (2)

Process uncertainty is introduced through variability in the parameters *L*_∞_ and *k* by using the same values as those generated for the growth model, giving 1000 values for each length. This model is referred to as the ‘Gislason’ model.

### Slicing length-based to age-based data

To convert the length-based stock and indices data to age-based a simple slicing method was used where each length-based observation is allocated to a corresponding age, based on the growth model, and aggregated accordingly (sums for abundances, abundance weighted means for the mean weights at length, means for natural mortality and maturity). The slicing was applied to the length-based stock data and each of the natural mortality models. Although the slicing method is deterministic, the length-based data was sliced by each of the 1000 sets of the growth parameters, thereby propagating the biological process uncertainty through to the age-based data.

The result of the slicing was two age-based stocks (one with the ‘0.4’ natural mortality model and one with the ‘Gislason’ natural mortality model, reflecting model uncertainty in the natural mortality assumptions), each with 1000 iterations in the data (reflecting process uncertainty in the growth and natural mortality assumptions). The indices of abundances were also sliced using the same growth model parameters giving three indices of abundances, each also with 1000 iterations.

### Model and estimation uncertainty in the stock assessment

The *a4a* statistical catch-at-age stock assessment model was used to assess both of the age-based stocks [9]. The *a4a* model requires setting up three submodels for the fishing mortality (the *fmodel*), the index catchability (the *qmodel*, one for each index) and recruitment (the *rmodel*). To introduce model uncertainty in the assessment, combinations of different submodels were used. Three *fmodel*s, three *qmodel*s and two *rmodel*s were used, giving a total of 18 stock assessment models (Table 3).

**Table 3:** The stock assessment model options for the 18 different stock assessment models.

SA model	fmodel	qmodel	rmodel	0.4	Gislason
1	age and year factors	age smoother	year smoother	711	825
2	spline on age and year	age smoother	year smoother	1000	990
3	logistic with year smoother	age smoother	year smoother	995	982 R
4	age and year factors	age and year smoothers	year smoother	789	748
5	spline on age and year	age and year smoothers	year smoother	998	995
6	logistic with year smoother	age and year smoothers	year smoother	961 R	984 R
7	age and year factors	logistic with year smoother	year smoother	769	747
8	spline on age and year	logistic with year smoother	year smoother	996 R	997
9	logistic with year smoother	logistic with year smoother	year smoother	926	990 R
10	age and year factors	age smoother	Ricker	867	629 R
11	spline on age and year	age smoother	Ricker	838 R	980
12	logistic with year smoother	age smoother	Ricker	1000	999
13	age and year factors	age and year smoothers	Ricker	841 R	582 R
14	spline on age and year	age and year smoothers	Ricker	875 R	977
15	logistic with year smoother	age and year smoothers	Ricker	998	1000
16	age and year factors	logistic with year smoother	Ricker	789	612
17	spline on age and year	logistic with year smoother	Ricker	972	983
18	logistic with year smoother	logistic with year smoother	Ricker	998	1000

The stock assessment model is made up of three submodels to model the fishing mortality (*fmodel*), survey catchability (*qmodel*) and recruitment (*rmodel*). The number of iterations that fitted successfully (out of 1000) for each natural mortality model choice are shown in the ‘0.4’ and ‘Gislason’ columns respectively. The presence of ‘R’ in the column indicates that the estimated stock was ultimately rejected for having bimodality in the estimated harvest rates in the final year, indicating instability in the model fit. For each stock assessment model the same *qmodel* was applied to each of the three indices of abundance. The degrees of freedom on the smoothers and the tensor splines was adjusted for the number ages in the data. The Ricker *rmodel* had a CV of 10% on the parameters.

The submodels were chosen to represent a reasonable spread across ‘model space’ for the stock and fishing fleets, with different patterns and assumptions underneath each option. The *fmodel* was either a linear model with factors on age and year, a smooth tensor spline over ages and years or a logistic curve over ages with a year smoother. The *qmodel* was either a combination of an age and year smoother or a logistic curve over ages with a year smoother. The same *qmodel* was applied to all three indices. The *rmodel* was either a smoother over years or a Ricker model [34]. Out of these submodels only the logistic curve (*fmodel* and *qmodel*) and Ricker (*rmodel*) impose a particular shape on the estimated data.

Each of the 18 stock assessment model combinations was used to assess each of the 1000 iterations (which represent process uncertainty) of the two stocks (which have either the ‘Gislason’ or ‘0.4’ natural mortality model). After fitting each iteration, estimation uncertainty was included by resampling the estimated model parameters from the reported variance in each fit.

No assumption was made about which of the submodel combinations is the most appropriate for the assessment given the data and no attempt was made to adjust the submodel parameter settings to achieve the ‘best’ fit for each iteration.

The output of the stock assessment stage was 36 model variants containing different stock assessment results, each with 1000 iterations. Each model variant represented an estimated stock with a different combination of the model uncertainties plus process and estimation uncertainty.

### Integrating across multi-model results with model averaging

In a traditional stock assessment process, only one of the estimated model variants would be selected as the single ‘best’ model (and would often only consider estimation uncertainty, not the combination of estimation and process uncertainty that exists here). Here, the results of the stock assessments are combined using a simple model averaging method based on the generalised cross-validation (GCV) score [35]. The GCV score is a measure of the predictive power of the model and can be used as a data-driven indicator of the quality of the fit [36]. The GCV score is estimated by an analytical expression, which makes it particularly suited for statistical catch-at-age models, as it doesn’t require a computer intensive leave-one-out procedure. The approach used in this study computed the GCV of the catch-at-age matrix only. Each model variant was assigned a weight based on the median GCV across its iterations. The lower the median GCV, the better the predictive power, on average, of the model variant. The inverse of the median GCV was used to weight each model variant so that variants with more predictive power had more weight assigned to them. The iterations within each model variant were assumed to be equally likely and were selected from at random.

The result of the model averaging was a single stock assessment with multiple iterations that incorporates process uncertainty in the growth and natural mortality parameters, natural mortality and stock assessment model uncertainty and stock assessment estimation uncertainty. This is in comparison to the original Gadget model used to assess the hake stock that did not include any sources of uncertainty.

## Results

### Process uncertainty in the growth parameters

**Fig 2:**
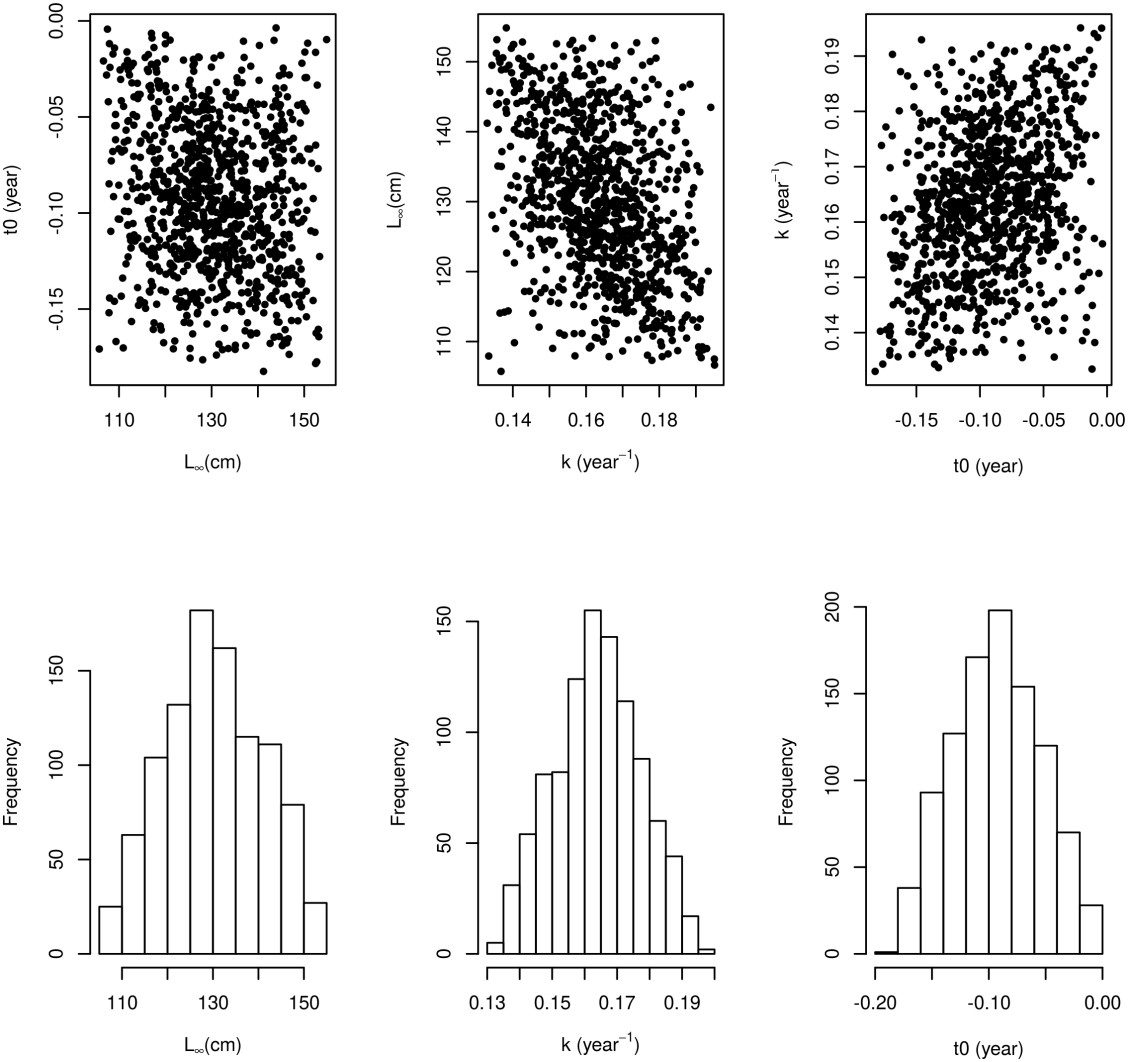
Including process uncertainty through the von Bertalanffy growth parameters. Top row: Pair wise scatter plots of 1000 samples of the von Bertalanffy growth parameters *L*_∞_, *k* and *t*0 that are used in the length-slicing and in the ‘Gislason’ natural mortality model. Bottom row: histograms showing the triangle marginals of the growth parameters. The spread of values in the plots reflects the process uncertainty in the parameter values.

**Fig 3:**
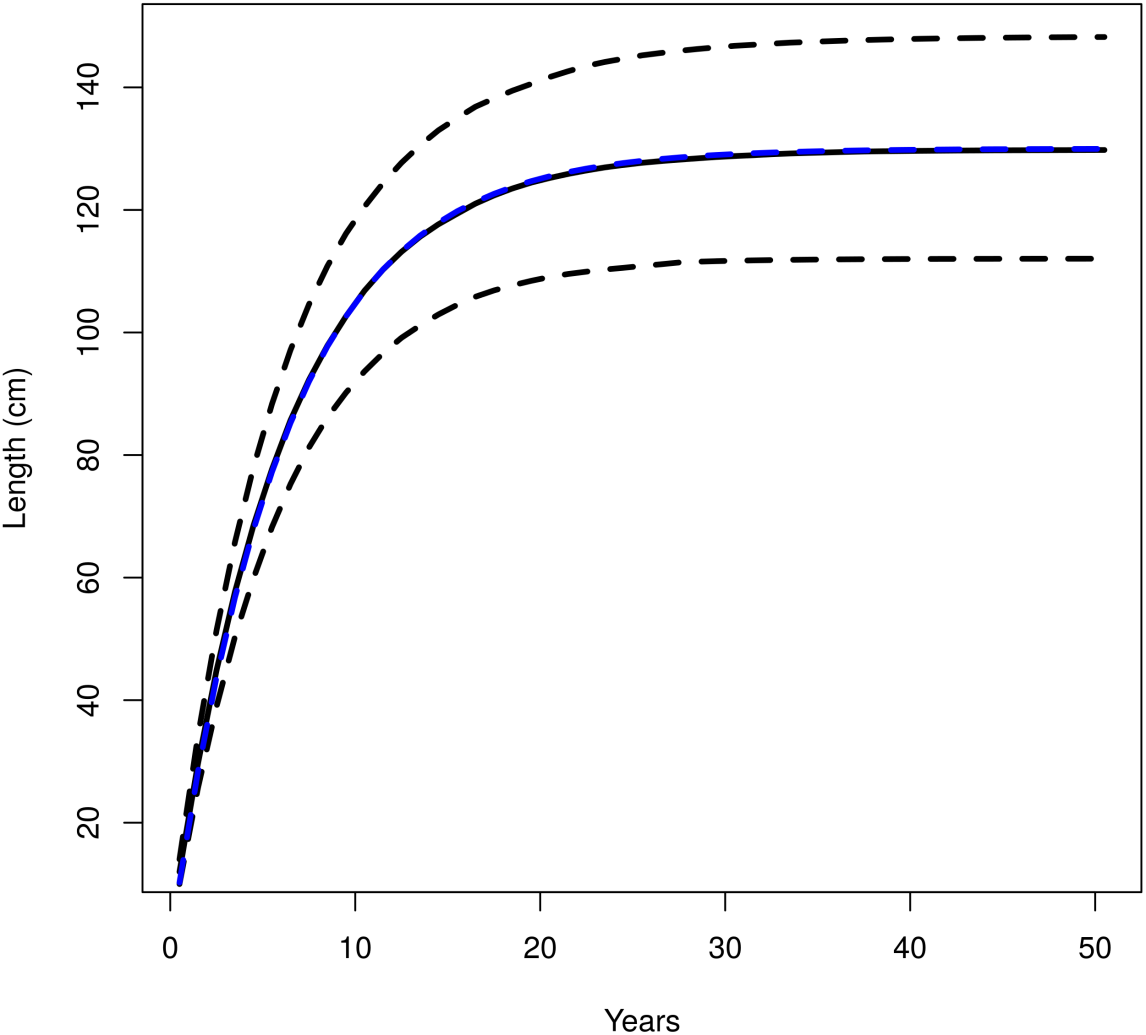
Variance in the von Bertalanffy growth curve resulting from the process uncertainty in the growth parameters *L*_∞_, *k* and *t*0. Median (solid black line) and 5% and 95% quantiles (dashed black lines). The deterministic growth curve using values for the growth parameters from the last ICES assessment (*L*_∞_ = 130, *k* = 0.164 and *t*0 = 0) is the blue, dashed line that runs through the median of the box plot.

The multivariate distribution of the von Bertalanffy growth parameters computed from Fishbase can be seen in Fig 2. *L*_∞_ and *k* are negatively correlated, *k* and *t*0 are positively correlated and *L*_∞_ and *t*0 have only a weak negative correlation. These samples are used in the length-slicing and in the ‘Gislason’ natural mortality model. The resulting uncertainty in individual size increases with age and the median value follows the growth curve of the most recent ICES assessment (Fig 3).

**Fig 4:**
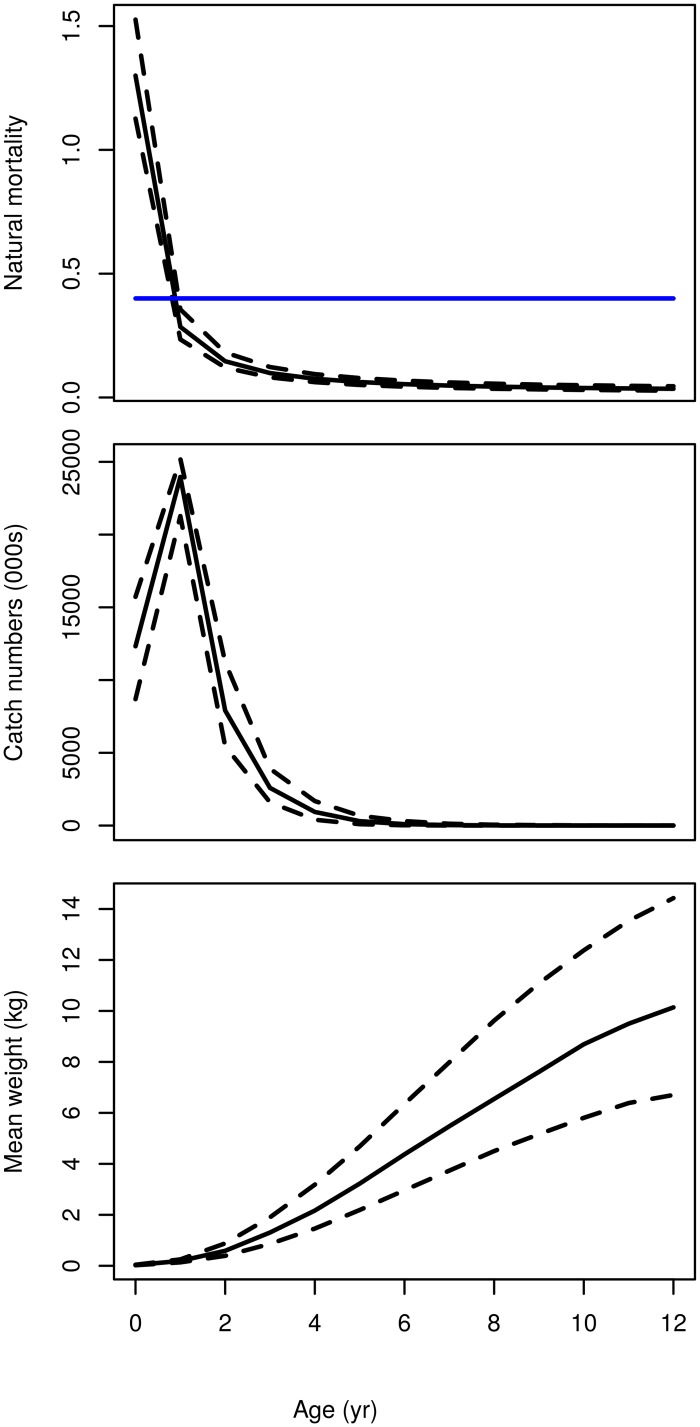
Example age-based stock data after the length-based data has been sliced using the uncertain von Bertalanffy growth parameters. Natural mortality, catch numbers and mean weights by age after slicing the length-based data. Median (solid line) and 5% and 95% quantiles (dashed line) are shown. The values are for the year 2012. Only ages up to 12 are shown for brevity. The two different natural mortality models are shown in the top panel. The ‘Gislason’ model is black and the ‘0.4’ model is blue. The variance in the ‘Gislason’ model represents the process uncertainty. The ‘0.4’ model has no process uncertainty and therefore no variance.

Example results of converting the length-based stock data to age-based data using the slicing method can be seen in Fig 4. The ‘Gislason’ model has higher values of natural mortality in the first age class than the ‘0.4’ model and also has uncertainty around the values (the ‘0.4’ model has no process uncertainty and therefore no variance). It can be argued that this is more biologically plausible than using the same values for all ages and the high variance reflects the high level of uncertainty in estimates of natural mortality in the early ages. The variance in the catch numbers in the younger ages is also very high reflecting high uncertainty in these ages. The variance in the mean weights at age increases as individuals get older, following the same pattern as the growth curve in Fig 3.

The uncertainty in the growth parameters meant that the age structures of the stock iterations could be different, i.e. some combinations of growth parameters resulted in much longer lived individuals than others. Therefore, each stock iteration had its own plusgroup (the last age that groups older ages) which was set at the age which contained 95% of the total catch biomass, averaged over the time series. The plusgroups ranged from 6 to 20 with a median value of 9.

### Stock assessment and natural mortality model uncertainty

Due to the level of process uncertainty, not all of the iterations of each estimated model variant fitted successfully. Iterations that did not fit successfully were removed. There was no other filtering to remove iterations that contained estimates that could be thought of as implausible (for example, there were several iterations with a mean fishing mortality greater than 8). However, some of the model variants had estimated values with multimodal distributions, particularly in the estimated harvest rates (catch biomass / stock biomass). This suggested that the fits of those model variants were unstable due to a combination of the different natural mortality models, stock assessment submodels and the process uncertainty. Hartigan’s dip test for bimodality was performed on the estimated harvest rates in the final year using the *diptest* package for R [37] to identify the unstable fits. It was found that 10 out of the 36 model variants had bimodal results. Due to the problems with fitting, these model variants were not considered to represent plausible scenarios and were rejected from the remainder of the analysis (Table 3). There did not appear to be any particular pattern to the rejected model variants, with all stock assessment submodels and both natural mortality models being components of the rejected variants.

The result was 26 fitted model variants, each of which can be thought of as a plausible combination of the different assessment and natural mortality models. Each variant had a minimum of 612 iterations, reflecting the process uncertainty in the growth and natural mortality parameters and the estimation uncertainty in the stock assessment fit.

**Fig 5:**
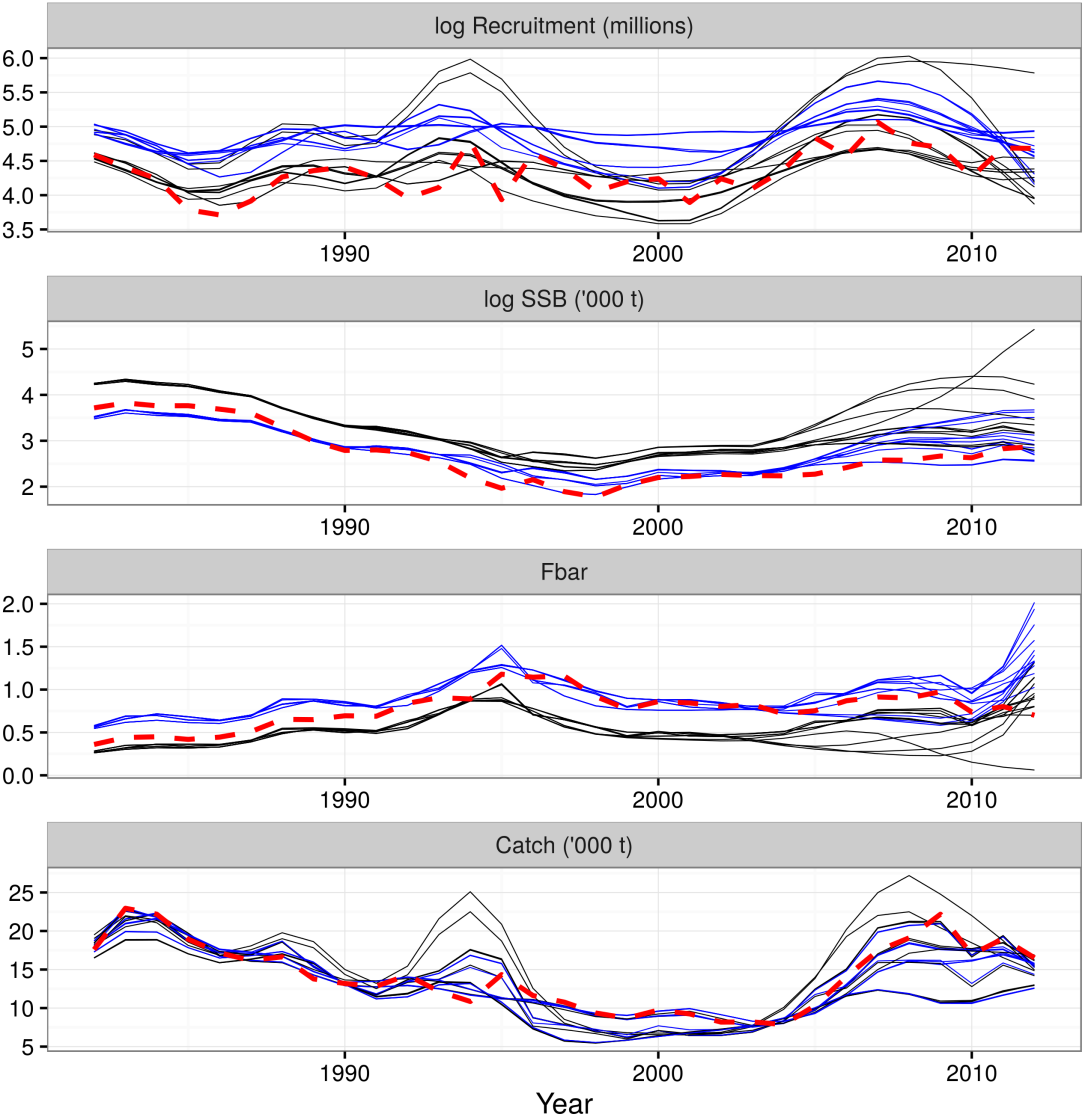
The impact of model uncertainty on the summary stock assessment results. Summary stock assessment results (recruitment, spawning stock biomass (SSB), mean fishing mortality (Fbar) and catch) from fitting a single iteration of the biological parameters with the 26 combinations of stock assessment and natural mortality models. This is equivalent to performing stock assessments without process or estimation uncertainty and only including model uncertainty. There are clear differences between the patterns and trends of the fits from each model, particularly in the most recent years. Note that the recruitment and SSB are shown on a log scale to allow the differences between the model results to be more visible. The recruitment, SSB and Fbar results can be broadly separated into two groups, driven by the natural mortality model. The ‘Gislason’ natural mortality model (blue lines) estimates higher recruitment and Fbar and lower SSB than the ‘0.4’ model (black lines). The results from the most recent ICES stock assessment are shown as the thick, dashed, red line.

Model uncertainty is not often considered in stock assessments other than attempting to find the single ‘best’ model within a suite of candidate models and discarding the other plausible models. Different stock assessment and natural mortality model combinations will obviously result in different stock assessment results. The impact of the model uncertainty can be illustrated by fitting each model combination with only a single iteration of the biological parameters (Fig 5). This ignores process and estimation uncertainty. There are clear differences in the patterns and trends in the results, particularly in the most recent years. For example, the mean fishing mortality in the final year ranges from 0.04 to 1.95. Models with the ‘Gislason’ natural mortality model all tend to have higher mean fishing mortality and recruitment but lower SSB than the models with ‘0.4’ natural mortality model, driven by the high natural mortality in the younger ages and low natural mortality in the older age.

**Fig 6:**
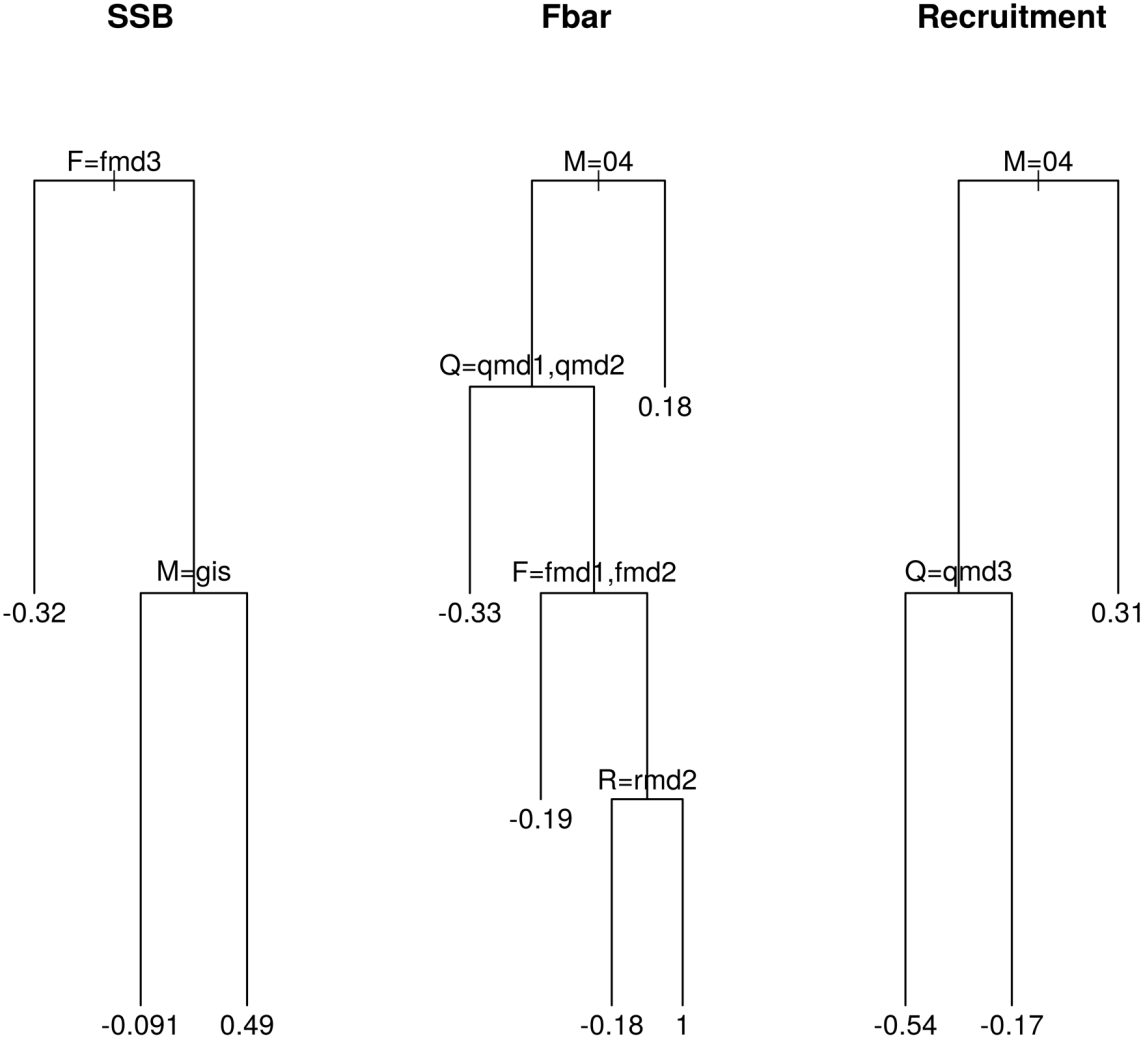
Regression trees showing which stock assessment and natural mortality model components had the biggest impact on the estimated stock assessment summary results. The summary stock assessment results are spawning stock biomass (SSB), mean fishing mortality (Fbar) and recruitment. The notation for *F*, *Q* and *R* refers to the submodel number in Table 3. For example, *Q = qmd1* means the second *qmodel* (the logistic model). *M* refers to the natural mortality model, either ‘0.4’ or ‘Gislason’. The numbers are the mean residuals from each model component on the logarithm of each summary measure.

The impact of the different natural mortality, fishing mortality, survey catchability and stock-recruitment model components on important fisheries variables (spawning stock biomass (SSB), recruitment and mean fishing mortality) was investigated using classification regression trees [38] to recursively partition the variables across the distinct model components. The analysis identifies the model components which have the biggest effect on the variable estimates (Fig 6). The analysis was carried out with the R package *rpart* [39].

With regards to SSB, the fishing mortality model was the most important, followed by the natural mortality model. For mean fishing mortality and recruitment the natural mortality model was the major factor, followed by the survey catchability model. The impact of the different model component on the estimates of these metrics demonstrate how important it is to account for uncertainty in model structure, particularly natural mortality.

### Model averaging

The process described here generated a suite of 26 fitted model variants. The final stage was to integrate the results from all of the variants using model averaging and produce a single set of results. The weightings for the simple model averaging method were based on the median GCV scores across the iterations of each fitted model variant. The minimum number of iterations in a single model variant was 612 (Table 3). To avoid overweighting any of the iterations in the model variants, the number of iterations used to build the model averaged results cannot be more than this minimum number. Therefore, 600 iterations were selected from across the 26 model variants based on their weight to construct a new ‘averaged’ set of results. Each iteration within a model variant was considered to be equally likely. Even though only 600 iterations were selected, the available iterations from each model variant ranged from 612 to 1000 (Table 3).

**Fig 7:**
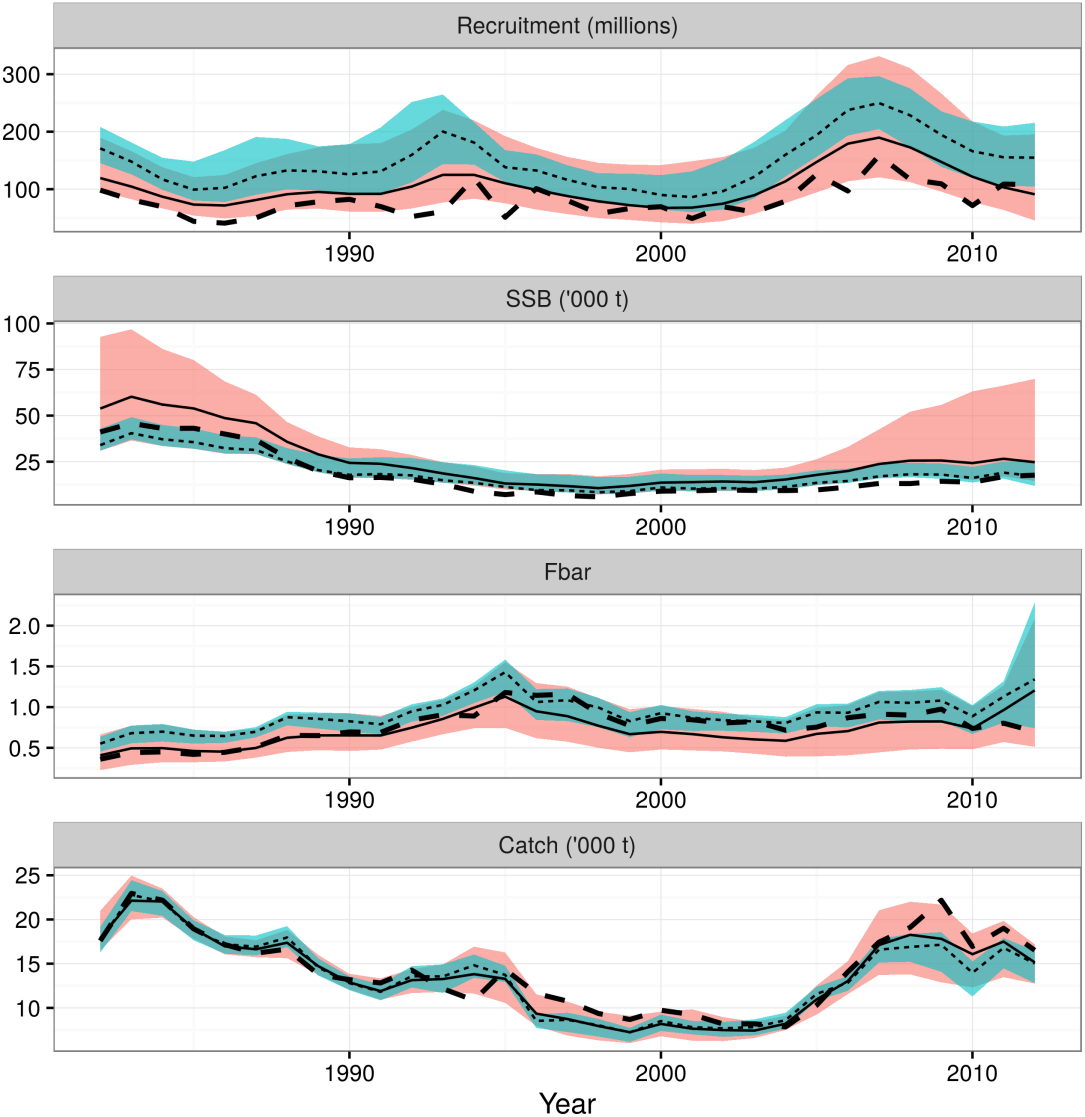
Comparing the model averaged results to the model variant with the lowest median GCV (the ‘best’ assessment). Summary metrics (recruitment, spawning stock biomass (SSB), mean fishing mortality (Fbar) and catch) from the model averaged results (red, sold line), the model with the lowest median GCV which can be used as an indicator of which model is ‘best’ (blue, thin dashed line) and the ICES assessment (thick dashed line). For the model averaged results and the GCV model, the lines show the medians and the ribbons show the 10 and 90% quantiles.

If we were interested in selecting only a single model, one method of selecting the ‘best’ estimated model is to select the one with the lowest median GCV score. That was stock assessment model 16 with the Gislason natural mortality model (Table 3). Comparing the ‘best’ GCV model and the model averaged results shows that both track the most recent ICES assessment, although recent SSB and recruitment estimates are higher (Fig 7). As expected, the variance in the single ‘best’ model is smaller than in the model averaged results, in particular on the uncertainty of the estimated fishing mortality. This is because the averaged results includes a greater range of model uncertainty. For example, the best GCV model only includes the Gislason natural mortality model, whereas the model averaged results integrate across both natural mortality models. It was shown above that the choice of natural mortality model has a large impact on the estimated fishing mortality.

**Fig 8:**
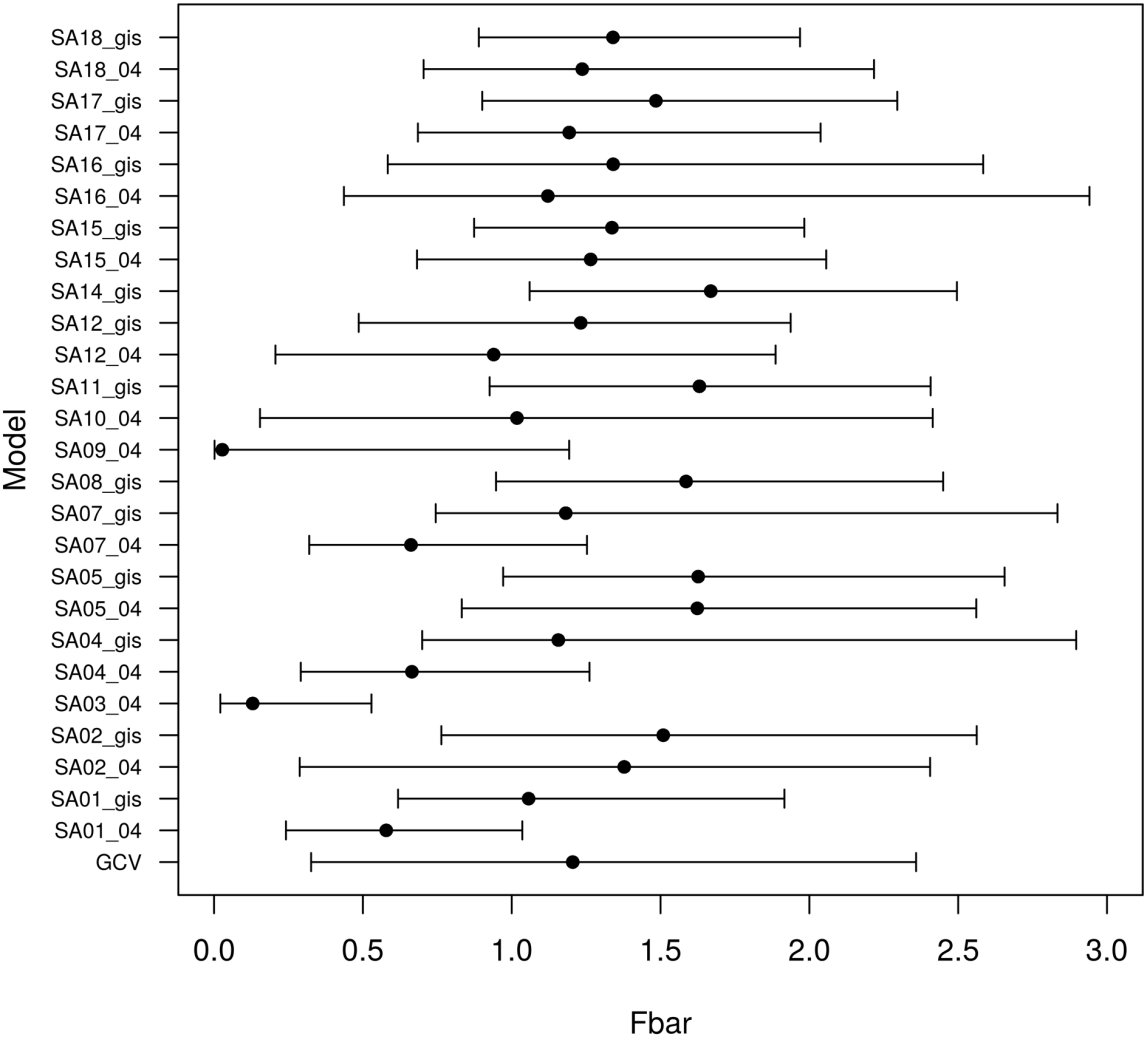
Distribution of the mean fishing mortality in the final year of the assessment for all model variants and model averaged results. The model labelling on the y-axis refers to the combination of the stock assessment submodels and the natural mortality model (see Table 3). *GCV* is the model averaged result using median GCV weighting. The points show the median value, the lines extend to the 5% and 95% quantile.

The effect of the model averaging process on the resulting variability in the results can be seen by looking at the estimated mean fishing mortality in the final year of the assessment for each of the individual fitted model variants and the model averaged results (Fig 8). The values for the model averaged results are taken from the full suite of 26 fitted model variants and therefore cover the full range of those values. As some of those fitted model variants contain fits that would be thought of as implausible, the model averaged results also contains some implausible results. For example, the maximum value of mean fishing mortality for the model averaged restuls is 8.46. However, the median value is 1.2 which is quite reasonable and the 90% quantile range is from 0.32 to 2.36. The averaged results contain all of the assumptions that were generated during the first step of the process. The results are therefore more robust than if only a single stock assessment result was selected from the model variants.

## Discussion

Estimating the status of a fish stock is challenging given the multiple sources and high levels of uncertainty that are present. Ensuring that the stock assessment process encompasses these uncertainties is important, given the key role the results often play in the advisory process. The ‘true’ states of the fishery, including the states of the biological and harvesting processes, are seldom known and so it is important that any provided advice is sufficiently robust to this uncertainty.

This paper presents an applied framework for considering and integrating multiple sources of uncertainty into the stock assessment process in three steps. The first step, conditioning, involves the generation of a suite of stocks that can be considered as representing plausible states of nature and carrying different assumptions. The second step, estimation, fits the different models to the data and adds estimation uncertainty to the results. Finally, the third step integrates the estimated stock assessments and their accompanying uncertainty into a single set of results. These results are a stochastic representation of the fleet and stock dynamics, that integrates across a wide range of assumptions and conditions.

The impact on advice will affect several processes like the estimation of biological reference points, forecasting, scenarios evaluation and/or risk analysis. A quantitative evaluation of all these processes was outside the scope of the paper. Nevertheless, we argue that by considering the several plausible states of nature instead of a single one, and integrating across several sources of uncertainty, our results span a large area of the model and parameters spaces, which makes any advice more robust to future natural conditions. When generating future scenarios the need to extrapolate outside current knowledge is reduced. Note that one could always expand the levels of uncertainty to amounts that would render the results useless for advice. However, the methodology presented here shows that it’s possible to include a large number of uncertainty factors while still keeping the results useful for advice.

One of the key strengths of the framework presented here is that each component of the conditioning step can be considered independently, with the uncertainty propagating between them. This means that the framework can be easily adapted to a particular fishery, making it applicable to stocks with varying levels of data availability. For example, biological uncertainty is included at an early stage in the framework by using a stochastic growth model to convert the length-based data to age-based. Here the growth parameters used the von Bertalanffy model with stochastic parameters taken from a multivariate distribution based on values from Fishbase combined with a t-copula and marginal triangle distributions. However, it would also have been possible to use an alternative growth model (e.g. Gompertz [23]), use a model that captures the sexual dimorphism of the hake stock, use alternative sources of data for the parameter values and use an alternative multivariate distribution (i.e using an alternative copula). Additionally, uncertainty could be included in processes that were deterministically modelled in this paper. For example, uncertainty could be included on the maturity ogive using a similar process as illustrated here. Non-stationary processes, for example temporal changes to the length-weight relationship, could also be included.

The use of a multivariate distribution to generate the parameters ensures that the relationships between the parameters are coherent resulting in plausible parameter sets. This is in contrast to other methods where life history parameter values are generated independently of each other, losing the relationship between them and resulting in a large number of rejected parameter sets e.g. [40].

The method for generating the growth parameters for the hake assessment presented here assumed stationarity in their distribution. However, there is evidence of non-stationarity in biological processes e.g. [41, 42]. It would be straightforward to include non-stationarity scenarios through trends in the marginal distributions of the growth parameters and the variance-covariance matrix. These scenarios could be used as additional plausible hypotheses when building the suite of stock assessment models.

Integrating across multi-model results has several advantages for the stock assessment process [7]. For example, the choice of natural mortality model was shown to have a strong impact on the estimates of recruitment and fishing mortality. This suggests that it is preferable to integrate across multiple natural mortality models instead of selecting a single model. By integrating across the results, there is no need to pick a single model from the suite of models. Instead more time can be spent on defining the initial suite, ensuring that the models are plausible and cover a wide range of ‘model space’ (to prevent models having similar characteristics, which when averaged across can give too much weight to the same type of model). This moves the focus of the assessment process away from model checking and model selection. Instead, designing the appropriate, plausible models becomes the most important task. Although only one model may be the most likely, the others still represent plausible ‘states of nature’ and contribute to the estimation of uncertainty about it. When only one model is selected, the assumptions behind the other models are rejected despite those assumptions also being plausible. Integrating over the results avoids the pitfalls of using a single model such as underreporting of variability, too narrow confidence intervals, overly optimistic tests of significance and potentially biased results [43]. This is demonstrated in the results, where selecting a single model based on the GCV resulted in lower variance in the summary stock assessment statistics than the model averaged stock.

This paper used model averaging to integrate over the results. It was not intended to offer a full description of how model averaging should be carried out and only a simple method is used. A key concern in model averaging is how to weight the models [8]. The most simple weighting method is to assign equal weights to each model. This assumes that all models are effectively equal in terms of plausibility. This is less selective about which models are drawn from than using a method based on model fit and should be used when there is no other criteria for selecting models, i.e. in the absence of further information all models are equally likely. Here, the weighting was based on the median GCV of each model which was taken to be a measure of how well that particular model fitted the data. Alternative methods based on measures of fit include using the Akaike Information Criterion (AIC) or the Bayesian Information Criterion (BIC) [44]. Use of the AIC was explored here but it was found that nearly all of the weight was put on only a single model variant and it was not pursued further. It is also possible to use qualitative weighting. For example, the International Whaling Commission combines the results from testing management procedures with model variants using qualitative weighting in a framework similar to the one presented here [45].

Structural uncertainty in the stock assessment models can also be a concern and can prove to be of greater magnitude than estimation uncertainty within a given model [46]. The simple model averaging approach used here is only applicable because the models have the same data and error assumptions meaning that objective (data-based) weighting is available. When models include different likelihood functions (e.g. if the suite of stock assessment models included VPA based models as well as SCA models) then the simple model averaging approach used here is not appropriate, restricting the structural uncertainty that can be considered. In this case more complicated model averaging methods can be used that include incorporating expert option, using machine learning and ensemble approaches, e.g. [46, 47].

The general approach presented here is flexible enough to be tailored to individual cases. However this flexibility means that it is possible to generate a suite of models that are not internally consistent or plausible. Integrated assessment models, which also attempt to include multiple sources of uncertainty, e.g. by estimating growth parameters within the stock assessment model fit, generate more consistent models. Nevertheless, the trade-off is an increase in the complexity of the model and the inclusion of correlation in the parameters space, with the inherent difficulties estimating the parameters.

Performing a stock assessment is a demanding task and using this framework may seem like placing additional burdens on stock assessors. However, we argue that this is not necessarily the case. A key part of the standard stock assessment approach is the need to perform extensive diagnostic model checks to select the single ‘best’ model. Using the framework presented here, more time can instead be spent on defining the initial suite of plausible models for each stock, allowing experts to focus more on the science. The software tools to implement this approach already exist and by taking advantage of modern computing facilities, fitting 1000s of iterations for many model variants is certainly possible within an operational time frame. What is needed is a change of perspective, away from selecting and defending a single ‘best’ model and towards stock assessments integrating multidisciplinary ideas to produce robust advice.

Stock assessments are often performed as part of a regular management process and in this paper we are concerned with generating a full assessment of the stock status, including estimates of abundance and fishing mortality at age. In terms of effective management, it has been demonstrated that relatively simple assessment models combined with appropriate harvest control rules can perform at least as well as conventional stock assessments [48]. This questions the need to estimate 100s parameters in a full stock assessment when only knowing how to to respond to a signal in a stock indicator may be sufficient. It has also been argued that stock assessment methods are too complicated and weaknesses in the underlying data and assumptions are neglected [49].

We argue that there will still always be a need to perform more detailed assessments of stock status. For example, to perform projections, fit a stock-recruitment relationship or to use the assessment to condition an operating model as part of an MSE, more than a simple assessment is required. [50] suggested that the ‘future trend will be to base management decisions on simple rules that are more often data-based rather than model-based while the complex models will serve primarily to evaluate the robustness of these decision rules’. The methods presented here fall into the ‘complex models’ category.

Capturing the full uncertainty of a natural system is considered to be almost impossible and not worthwhile when the costs and benefits are taken into account. One solution is to divide the process into small pieces and deal with each one of them as required. However, each subprocess may be described in different ways, leading to uncertainty about which scenario, if any, is ‘correct’. Generating many plausible scenarios can also easily generate a large amount of results, creating problems downstream when attempting to keep an eye on the important messages without being overwhelmed. The method presented in the paper tries to navigate between these two problems and present operational solutions for integrating important sources of uncertainty into our perception of fish stock exploitation.
